# The efficacy and adverse events of bevacizumab combined with temozolomide in the treatment of glioma: a systemic review and meta-analysis of randomized controlled trials

**DOI:** 10.3389/fmed.2024.1419038

**Published:** 2024-07-02

**Authors:** SiYao Wei, LanYin Chang, Yi Zhong

**Affiliations:** ^1^Department of Neurology, The Affiliated Hospital of Southwest Medical University, Luzhou, China; ^2^Department of Otolaryngology Head and Neck Surgery, The Affiliated Hospital of Southwest Medical University, Luzhou, China; ^3^Department of Cardiology, The Affiliated Hospital of Southwest Medical University, Luzhou, China

**Keywords:** glioma, bevacizumab, temozolomide, efficacy, adverse events

## Abstract

**Objectives:**

To assess the efficacy and adverse events of bevacizumab (BEV) combined with temozolomide (TMZ) in the treatment of glioma.

**Materials and methods:**

Randomized controlled trials (RCT) involving BEV combined with TMZ in the treatment of glioma were searched using PubMed, Embase and Cochrane library, and a comprehensive meta-analysis was conducted. The primary outcomes were overall survival time (OS) and progression-free survival time (PFS), and the secondary outcome was adverse events. Researchers conducted literature screening, data extraction and quality assessment according to inclusion and exclusion criteria. RevMan 5.3 software was used for meta-analysis.

**Results:**

A total of 8 prospective RCTs of 3,039 cases were included in the meta-analysis. Meta-analysis showed that compared with TMZ alone, BEV combined with TMZ could significantly improve PFS, OS and complete remission rate (CR). A total of 6 studies reported related adverse events, mainly including thrombocytopenia, neutropenia, leukopenia, anemia and fatigue. Combination therapy may have more adverse events but no serious consequences.

**Conclusion:**

The combination of BEV and TMZ had a better therapeutic effect on glioblastoma, significantly prolonged the survival time of patients and improved the quality of life. However, some patients are afflicted with the adverse events of combination therapy, and subsequent studies should continue to conduct larger, multi-center RCTs to confirm the findings and explore in depth how to minimize and manage adverse events effectively.

## Introduction

Glioma originates from glial cells and is a common primary intracranial tumor in adults, which is characterized by high malignancy and invasive growth. Glioma accounts for about one-fifth of all central nervous system tumors and 80% of malignant central nervous system tumors, of which glioblastoma has the highest incidence in malignant central nervous system tumors, accounting for 46.1% (1). Glioblastoma is strongly associated with neurological deterioration, decreased functional independence, and quality of life (2). At present, surgical resection can be used for the treatment of glioblastoma, but due to the unclear boundary of the tumor and difficulty in completely removing the tumor, the risk of tumor recurrence is very high (3). Angiogenesis is a prominent pathological feature of glioblastoma and is mainly attributed to autocrine and paracrine secretion of vascular endothelial growth factor-A (VEGF-A), further up-regulating the VEGF signal transduction pathway, leading to its overexpression (4). Based on the results of clinical trials from European Cancer Research and Canadian National Cancer Research Center, TMZ has a therapeutic effect for glioblastoma, but the long-term survival rate of patients remains low and new treatment strategies still need to be explored (5). For cancer treatment, it is usually difficult to obtain an ideal therapeutic effect by using a single drug. Moreover, TMZ alone for glioma treatment can also cause the drug resistance (6). Some investigators also agree with this view that combination therapy is needed to improve the overall response rate of glioblastoma and reduce the drug resistance (7–9).

BEV is a monoclonal antibody targeting vascular endothelial growth factor, which can inhibit angiogenesis and continuously control tumor growth, so it has been widely used in the treatment of rectal cancer and lung cancer (10). Previous studies have shown that antiangiogenic agents have an inhibitory effect on glioblastoma (11). For the treatment of glioblastoma, BEV can bind to its blood circulation target, change the kinetic relationship between ligand binding to endothelial cells and down-regulate angiogenic signals, so as to achieve the purpose of inhibiting tumor growth (12). Therefore, BEV is a relatively suitable drug for glioma treatment in combination with TMZ.

At present, FDA has approved the combination of BEV and TMZ for the treatment of glioma, however, the results on the efficacy and safety of this treatment regimen are not completely consistent in recent years (13, 14). Some scholars believe that the combination of BEV and TMZ may increase the incidence of adverse reactions in patients, and its disadvantage is more obvious (15, 16). Given this situation, this meta-analysis was conducted to assess the efficacy and adverse events of BEV combined with TMZ in the treatment of glioma and provide evidence-based medicine basis for the rational use of this treatment strategy in clinical.

## Materials and methods

### Search strategy

This meta-analysis was conducted according to the Preferred Reporting Items for Systematic Reviews and Meta-Analyze guidelines (PRISMA). Several databases (PubMed, Embase, Cochrane Library and other databases) were retrieved as of February 2024 to extract study data, the literature language was limited to English. Primary search terms included (“BEV” OR “Bevacizumab”) AND (“Glioma” OR “Glioblastoma”) AND (“Randomized controlled trial” OR “RCT”). No restrictions were imposed on race, age, sex, or other factors during the search.

### Inclusion and exclusion criteria

The literature screening process was performed by two investigators in accordance with the inclusion and exclusion criteria, if there are discrepancies between the two investigators, a third investigator will decide whether to include the study. Inter-rater reliability was assessed using Cohen’s kappa coefficient. Inclusion criteria were as follows: (1) Patients with definitive diagnosis of gliomas; (2) BEV and TMZ were used in the treatment regimen; (3) Study type is RCT; (4) Number of subjects included in the study >20; (5) All studies had clear outcome measures, including overall survival and progression-free survival, etc. Exclusion criteria were as follows: (1) The types of studies were review or case report; (2) Study subjects were animals; (3) Patients with other tumor diseases; (4) Study results unclear, no raw data available.

### Data extraction

After literature screening according to inclusion criteria, study data extraction was performed by researcher. The following information was extracted from each trial: (1) published year of trial; (2) the name of first author; (3) number of participants included in trial; (4) median age of patients; (5) gender of participating patients; (6) study intervention measures; (7) study primary outcome; (8) related adverse events. All data included in this review were obtained from peer-reviewed published studies.

### Quality assessment

Bias risk assessment tool recommended by Cochrane was used to analyze the bias risk of the included RCTs. Each of the following areas was evaluated at the trial level: hidden random sequence generation and allocation (selection bias); blind method of participants and personnel (performance bias); blind method of outcome evaluation (test bias); incomplete result data (attrition bias); selective results report (report bias). And other deviations (for example, baseline imbalance, early termination of trials, industry or funding deviations, missing sample size calculations or other defects in statistical analysis). Each potential source of deviation is rated as “high,” “low” or “unclear” risk.

### Statistical analysis

The hazard ratio (HR) and their respective 95% confidence intervals (CI) were evaluated as a measure of the effectiveness. If the study reports adjusted and unadjusted hazard ratios, the adjusted hazard ratios are used for primary analysis. For the binary classification results, the odds ratio (OR) and its respective 95% confidence intervals are regarded as effects. RevMan 5.3 and STATA 17.0 software were used for meta-analysis, and forest plots were drawn for analysis. *I*^2^ statistics were used for heterogeneity test. If there is no significant heterogeneity between studies (*I*^2^ ≤ 50%, *p* < 0.05), the fixed effect model was used to merge the data. If there is significant heterogeneity between studies (*I*^2^ > 50%, *p* ≥ 0.05), random effects model was used to merge the data. Publication bias was evaluated by funnel plot, and sensitivity analysis was assessed by leave-one-out method.

## Results

### Eligible studies

**Figure 1:**
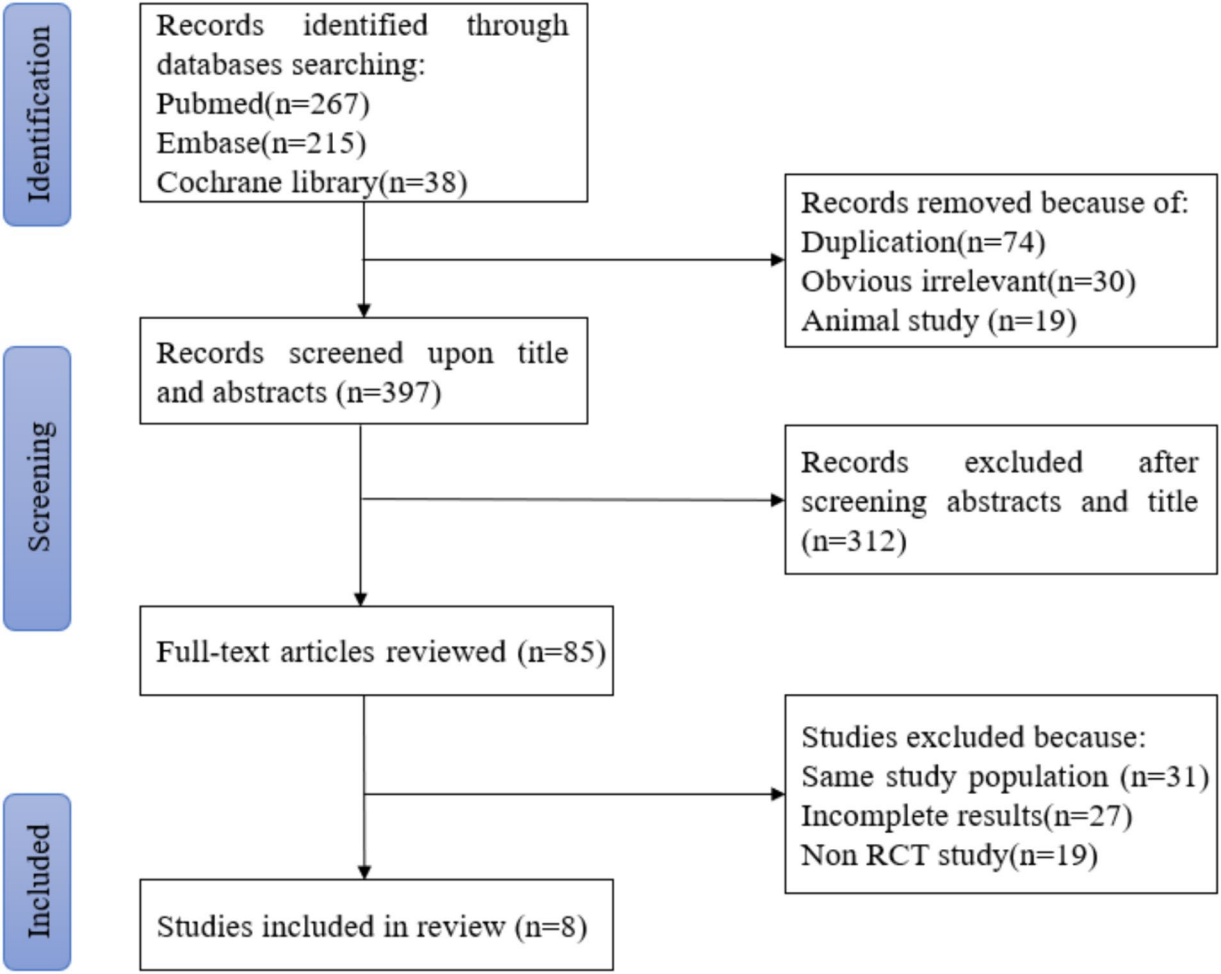
PRISMA study diagram of literature search and study selection.

**Figure 2:**
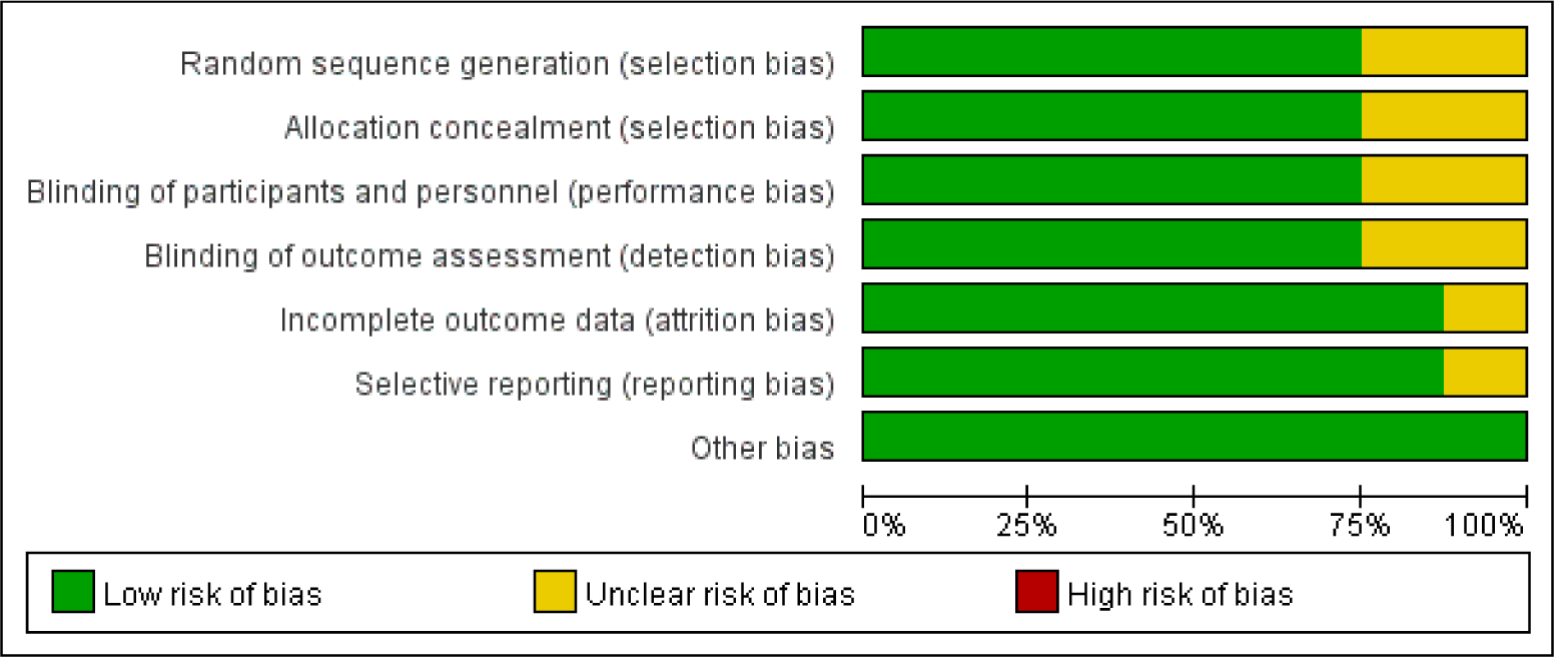
The risk of bias graph.

**Figure 3:**
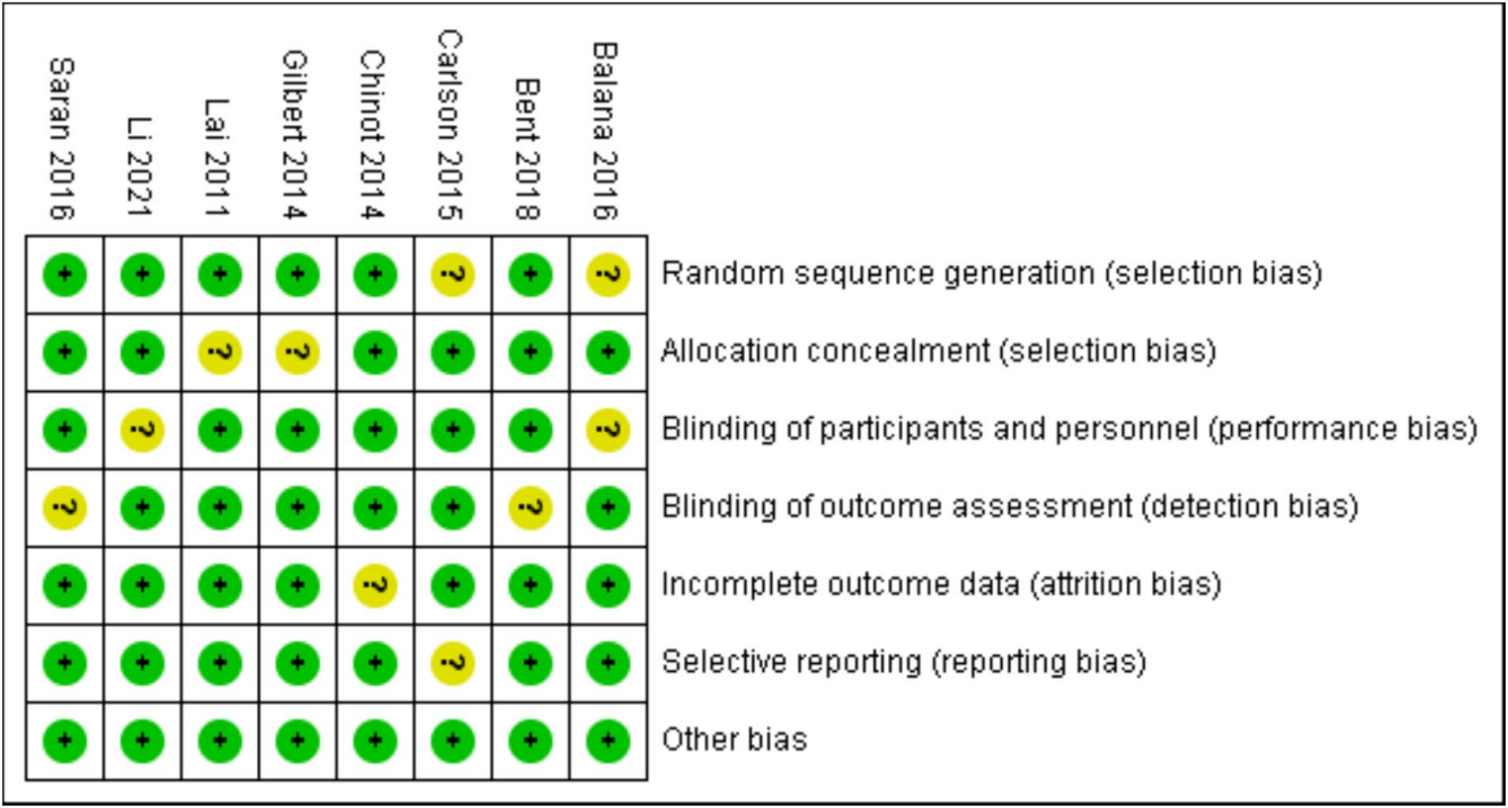
The risk of bias summary.

According to the retrieval method mentioned above, a total of 520 potentially relevant studies were assessed. The detailed steps were shown in Figure 1. After the selection procedure, eight articles were included (17–24), with a total of 3,039 patients with glioma. Specific quality assessment results of eligible studies were illustrated in Figures 2, 3, the basic characteristics of the included studies are listed in detail in Table 1.

**Table 1:** Study characteristics of included studies.

Author	Year	Study type	Number of participants		Median age		Male/female		Interventions		Adverse event (Grade 3-4)		Outcomes
			Experimental	Control	Experimental	Control	Experimental	Control	Experimental	Control	Experimental	Control	
Li (12)	2021	RCT	51	51	54.2 (45–77)	52.8 (42–73)	29/22	26/25	BEV + TMZ	TMZ	7	2	a,b,c
Bent (11)	2018	RCT	78	77	44.6 (33.9–53.8)	43.1 (34.5–49.0)	57/21	45/32	BEV + TMZ	TMZ	45	17	b,c
Balana (15)	2016	RCT	48	45	62.9 (43–75)	62 (36–75)	31/17	25/20	BEV + TMZ	TMZ	24	12	a,b,c
Saran (16)	2016	RCT	461	450	57 (20–84)	56 (18–79)	285/176	290/160	BEV + TMZ	TMZ	297	166	b,c
Carlson (14)	2015	RCT	30	26	56.5 (31–78)	60.5 (25–77)	17/13	16/10	BEV + TMZ	TMZ	–	-	b,c
Chinot (18)	2014	RCT	458	463	57 (20–84)	56 (18–79)	282/176	298/165	BEV + TMZ	TMZ	306	237	b,c
Gilbert (13)	2014	RCT	312	309	58 (19–82)	60 (18–87)	178/134	194/115	BEV + TMZ	TMZ	138	96	b,c
Lai (17)	2011	RCT	70	110	57.4 (31.3–75.8)	59.4 (20.5–90)	39/31	70/40	BEV + TMZ	TMZ	–	-	b,c

RCT, randomized control trial; BEV, bevacizumab; TMZ, temozolomide. a, CR (Complete response rate); b, PFS (Progression-Free Survival); c, OS (Overall Survival).

### Efficacy

**Figure 4:**
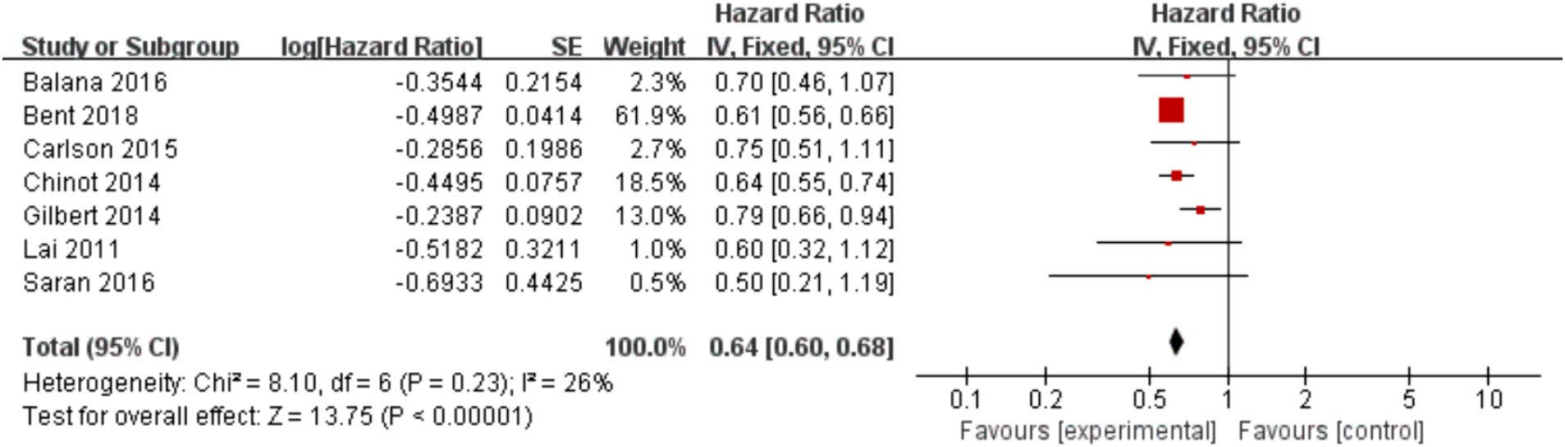
Forest plot of progression-free survival.

**Figure 5:**
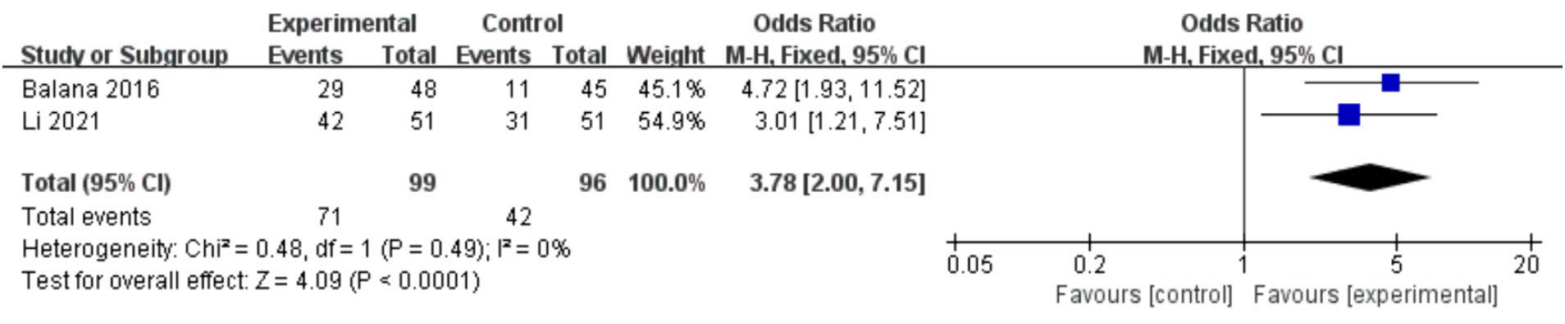
Forest plot of overall complete remission rate.

**Figure 6:**
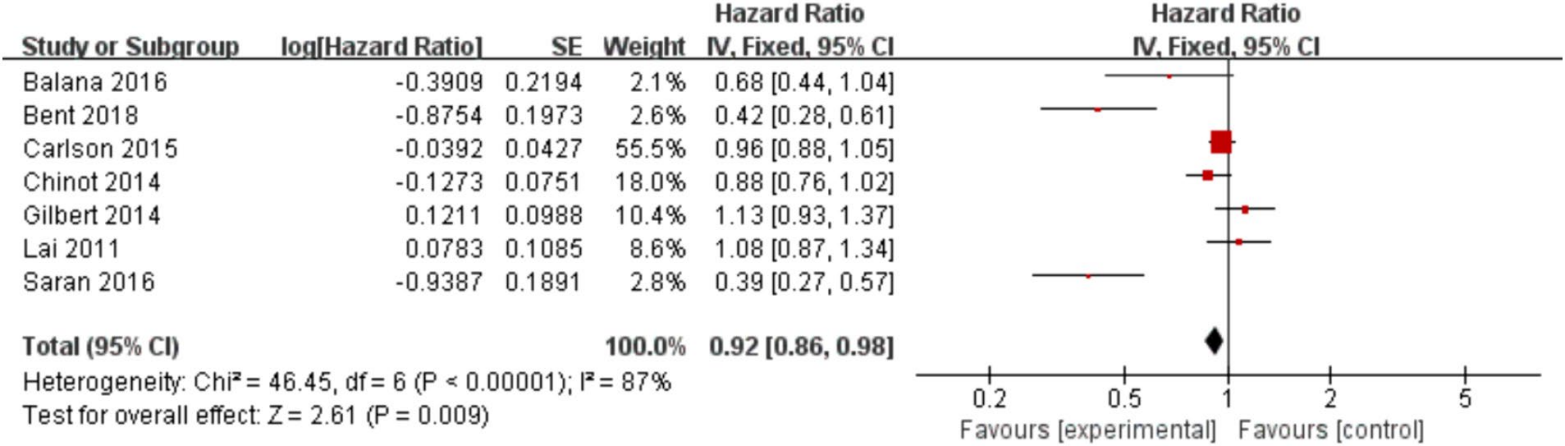
Forest plot of overall survival.

In the 8 included studies, 2 trials reported CR as the primary efficacy measure, and 7 trials reported PFS and OS as the primary efficacy measure. Meta-analysis of the fixed-effect model showed that PFS was significantly longer in the experimental group than in the control group [HR = 0.64, 95%CI (0.60, 0.68), *p* < 0.0001, Figure 4], and that CR was significantly increased in the experimental group than in the control group [OR = 3.78, 95%CI (2.00, 7.15), *p* < 0.0001, Figure 5]. Random-effect model showed that OS was significantly longer in the experimental group than in the control group [HR = 0.64, 95%CI (0.60, 0.68), *p* < 0.0001, Figure 6]. The results of the study demonstrated that the combination of BEV and TMZ was more effective than TMZ alone.

### Adverse events

**Figure 7:**
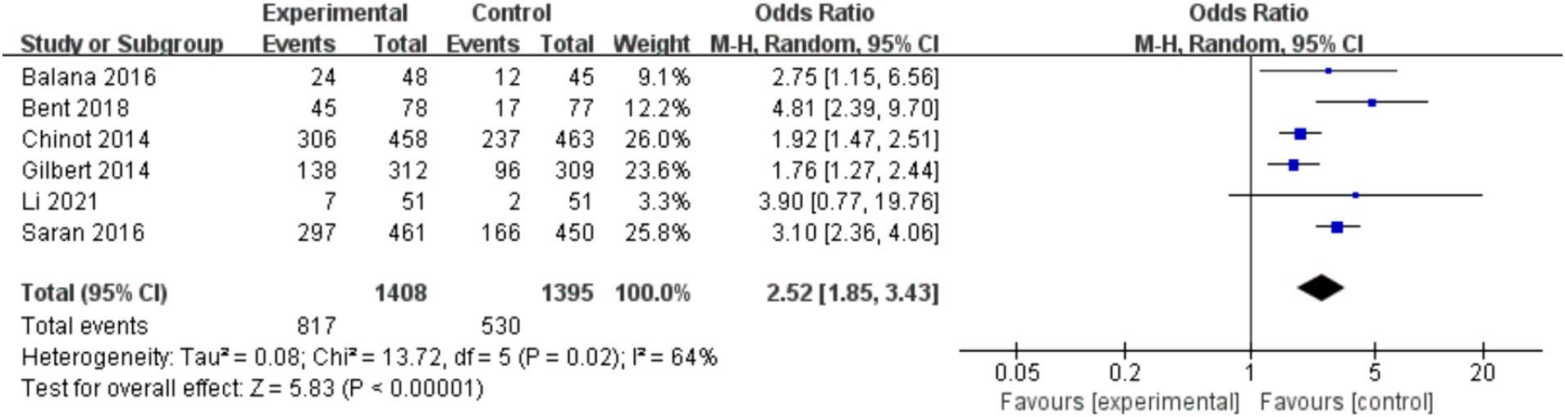
Forest plot of adverse events.

Among the included studies, six studies reported adverse events of BEV combined with TMZ in the treatment of glioblastoma, mainly including leukopenia, anemia, thrombocytopenia, neutropenia and fatigue. Meta-analysis of grade 3 or higher adverse events showed that the incidence of adverse events in the experimental group was higher than that in the control group (*p* < 0.000 01), as shown in Figure 7.

### Publication bias

**Figure 8:**
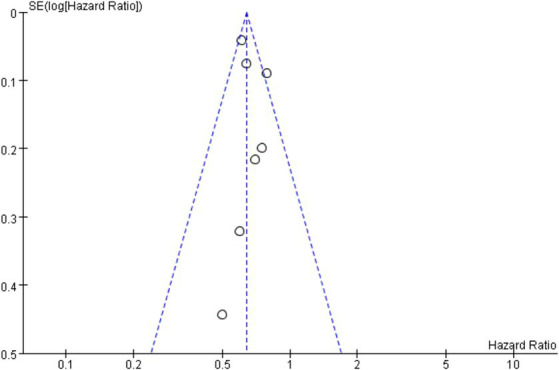
Funnel plot of overall survival.

In this study, overall survival is the primary outcomes, so OS was used for publication bias risk analysis. According to the results of funnel plot, there was a possibility of publication bias in this study, and funnel plot is shown in Figure 8.

### Sensitivity analysis

**Figure 9:**
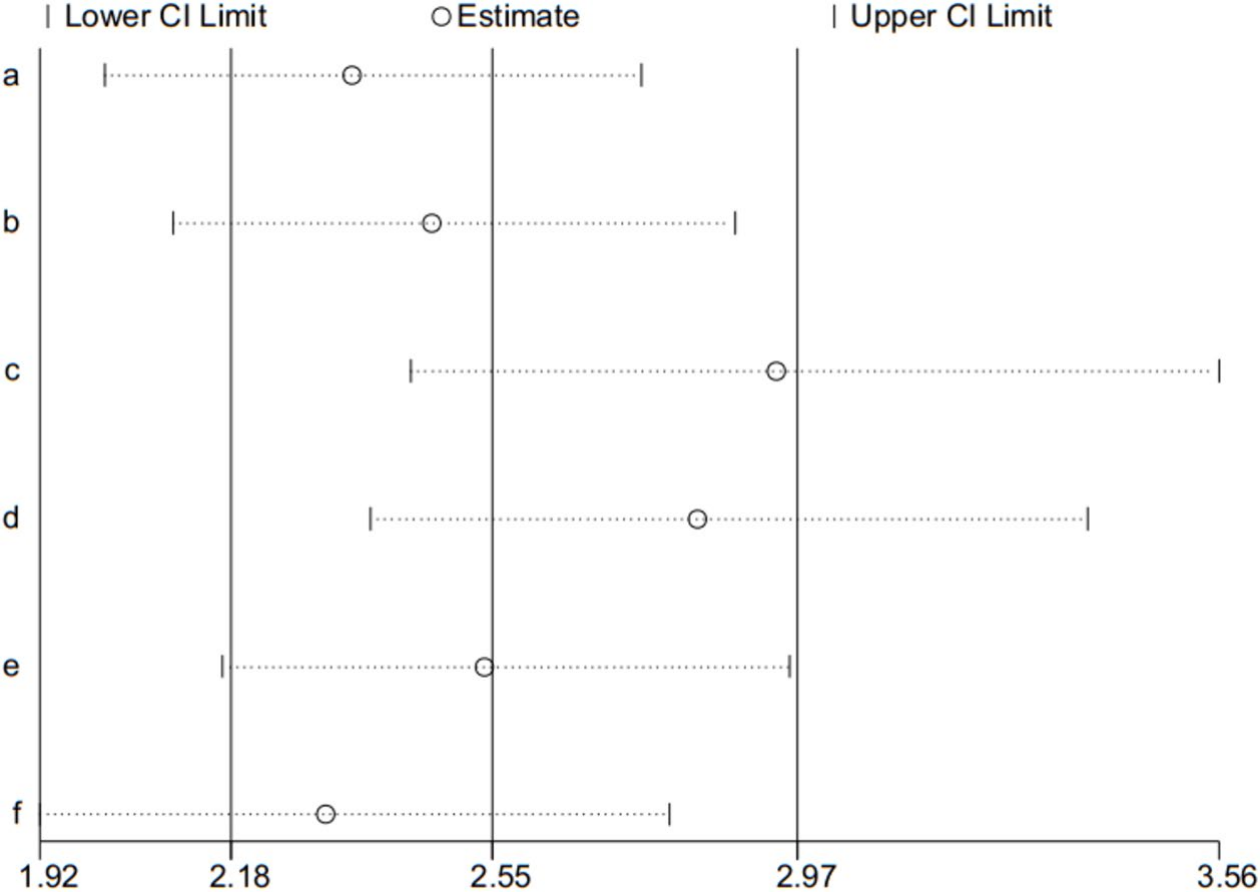
Sensitivity analysis of adverse events.

Adverse event is the second outcome, and was used for the sensitivity analysis. Sensitivity analysis was assessed by the leave-one-out method, the results presented that pooled effect changed after removing the included articles one by one (shown in Figure 9).

## Discussion

Glioma is the most common primary brain tumor, and its pathogenesis is not clear. Two known risk factors are exposure to high doses of ionizing radiation and genetic mutations. According to the WHO grading system, gliomas are divided into grades I-IV, grades I and II are low-grade gliomas, and slow tumor growth has a better prognosis through surgery; grades III (anaplastic astrocytoma) and IV (glioblastoma) are high-grade gliomas, of which glioblastoma patients with the highest degree of malignancy still have a median survival of less than 15 months even if the most aggressive treatment is used. The current standard of care for glioblastoma is to remove tumor cells as much as possible, followed by radiotherapy, chemotherapy, and adjuvant TMZ chemotherapy (25). Despite aggressive treatment, survival rates have not significantly improved, and most patients are prone to relapse, seriously affecting quality of life. In the treatment of glioblastoma, the basis of chemotherapy is to inhibit the division of rapidly growing cells, which is characteristic of cancer cells, but it also affects the metabolism of normal cells in the body and presents unique side effects of chemotherapy (26). The emergence of targeted drugs targeting cellular receptors, key genes, and regulatory molecules has brought new directions for the treatment of glioblastoma (27).

BEV is the first monoclonal antibody approved for marketing, which can competitively bind to vascular endothelial growth factor receptor, block VEGF-mediated angiogenesis, help inhibit angiogenesis of tumor tissue and weaken invasive ability, and has been approved for the first-line treatment of recurrent glioblastoma (28, 29). They can induce cancer cell death by blocking biological transduction pathways or specific cancer proteins, or specifically deliver chemotherapeutic agents to cancer cells to minimize adverse side effects (30–32).

Our results showed that the combination regimen significantly prolonged PFS in glioblastoma patients and reduced the hazard ratio of progression-free disease by 36%, indicating that this combination regimen was significantly effective. In addition, BEV combined with TMZ resulted in superior CR and OS compared with TMZ alone, which is also a reflection of the treatment effect, and there may be a synergistic effect between TMZ and BEV. The growth of tumor cells is inseparable from the blood supply. The anti-angiogenic effect of BEV has a positive effect on inhibiting the growth of tumor cells. At the same time, the inhibitory effect of TMZ on cellular DNA replication greatly controls the regeneration and division of tumor cells, which may be the mechanism involved in the antitumor growth of the combination regimen. BEV and TMZ cooperate with each other and bring better efficacy to glioma patients. Another similar study showed that BEV combined with chemotherapeutic drugs could significantly improve the overall survival rate of patients (33). As early as 2007, a Duke University trial of BEV combined with irinotecan in the treatment of recurrent malignant glioma showed that BEV combined with other drugs could lead to better therapeutic benefits (34). Subsequently, various studies combined BEV with multiple drugs to explore the optimal drug dose and frequency of treatment (35–37). This suggests that BEV has great potential for the treatment of glioblastoma, combining with TMZ in the treatment of glioma results in a stronger therapeutic effect. Combination therapy can bring longer survival time and better quality of life to glioma patients and has crucial clinical value.

In terms of adverse events, the experimental group had a higher incidence of adverse events than the control group, which increased the risk of patients to some extent. The emergence of this phenomenon may be related to the combination of drugs. TMZ inhibits the growth of tumor cells by inhibiting cellular DNA replication, but it also damages the DNA of normal cells, resulting in normal cell death (38, 39). And the metabolites of TMZ are small in size, easily absorbed, and have high blood–brain barrier permeability, which may be a relevant mechanism leading to the development of adverse events (40). Although BEV as an antiangiogenic agent combined with TMZ can significantly reduce the growth of tumor cells, it also interferes with the blood metabolism of normal tissues, and its clinical application value is still controversial (41). Previous studies have shown that anti-angiogenic agents are more effective than chemotherapy alone, but there are also some adverse effects, such as anemia and leukopenia (42). In our study, drug-related adverse reactions also emerged, however, these adverse events were controllable and tolerable, and the adverse events were effectively controlled after regular drug intervention in patients. Although the adverse events can be effectively controlled, it will also affect the quality of life of patients. In addition, the sensitivity analysis showed that the incidence of adverse reactions is not very stable, which may be related to the different ways in which adverse reactions were reported in included studies. The subsequent further study should focus on the control of adverse events of BEV combined with TMZ to avoid the occurrence of adverse events as far as possible. Future trials should use standardized reporting criteria, such as the Common Terminology Criteria for Adverse Events (CTCAE), to ensure consistent and comprehensive reporting of adverse events.

The results showed that there were possibilities of heterogeneity and publication bias in this study, which may be related to the following factors. Articles included in this study had a large chronological span, with a maximum span of 10 years, and there were large differences in the number of patients between studies, which may have contributed to the heterogeneity of the study results. In addition, although the included studies used combination therapy versus monotherapy, there were slight differences in the dose and frequency of the drugs used in each study, which may have led to heterogeneity of the meta-analysis. Furthermore, in performing the literature search, we set strict inclusion criteria and did not search the gray literature. All study data came from published articles and did not contact investigators to obtain unpublished data.

### Limitations

Although this study evaluates combination regimens from multiple aspects, there are still some limitations. Firstly, the articles included in this study were RCTs, resulting in a small number of included articles, which may generate heterogeneity and weaken the reliability of the study results. Secondly, the start time of included studies spans a large span and may have heterogeneity on the results. Lastly, some included studies did not report the occurrence of adverse events, may lead to less comprehensive results for adverse events.

## Conclusion

This study showed that the combination of BEV and TMZ had a better therapeutic effect on glioblastoma, significantly prolonged the survival time of patients and improved the quality of life. However, some patients are afflicted with the adverse events of combination therapy. Subsequent studies should continue to conduct larger, multi-center RCTs to confirm the findings and explore in depth how to minimize and manage adverse events effectively. In addition, optimal dosing and scheduling of BEV and TMZ should also be investigated.

## Data availability statement

The original contributions presented in the study are included in the article/supplementary material, further inquiries can be directed to the corresponding author.

## Author contributions

SW: Writing – review & editing, Writing – original draft, Conceptualization. LC: Writing – review & editing, Writing – original draft, Data curation. YZ: Writing – review & editing, Supervision, Software, Project administration, Methodology, Investigation, Formal analysis.
